# Identification of *Leptospira* and *Bartonella* among rodents collected across a habitat disturbance gradient along the Inter-Oceanic Highway in the southern Amazon Basin of Peru

**DOI:** 10.1371/journal.pone.0205068

**Published:** 2018-10-09

**Authors:** Valerie Cortez, Enrique Canal, J. Catherine Dupont-Turkowsky, Tatiana Quevedo, Christian Albujar, Ti-Cheng Chang, Gabriela Salmon-Mulanovich, Maria C. Guezala-Villavicencio, Mark P. Simons, Elisa Margolis, Stacey Schultz-Cherry, Víctor Pacheco, Daniel G. Bausch

**Affiliations:** 1 U.S. Naval Medical Research Unit No. 6, Callao, Peru; 2 St. Jude Children’s Research Hospital, Memphis, Tennessee, United States of America; 3 Pontificia Universidad Católica del Peru, Lima, Peru; 4 Universidad Nacional Mayor de San Marcos, Museo de Historia Natural, Lima, Peru; 5 Tulane University School of Public Health and Tropical Medicine, New Orleans, Louisiana, United States of America; University of California Davis, UNITED STATES

## Abstract

**Background:**

The southern Amazon Basin in the Madre de Dios region of Peru has undergone rapid deforestation and habitat disruption, leading to an unknown zoonotic risk to the growing communities in the area.

**Methodology/Principal findings:**

We surveyed the prevalence of rodent-borne *Leptospira* and *Bartonella*, as well as potential environmental sources of human exposure to *Leptospira*, in 4 communities along the Inter-Oceanic Highway in Madre de Dios. During the rainy and dry seasons of 2014–2015, we captured a total of 97 rodents representing 8 genera in areas that had experienced different degrees of habitat disturbance. Primarily by using 16S metagenomic sequencing, we found that most of the rodents (78%) tested positive for *Bartonella*, whereas 24% were positive for *Leptospira*; however, the patterns differed across seasons and the extent of habitat disruption. A high prevalence of *Bartonella* was identified in animals captured across both trapping seasons (72%–83%) and the relative abundance was correlated with increasing level of land disturbance. *Leptospira*-positive animals were more than twice as prevalent during the rainy season (37%) as during the dry season (14%). A seasonal fluctuation across the rainy, dry, and mid seasons was also apparent in environmental samples tested for *Leptospira* (range, 55%–89% of samples testing positive), and there was a high prevalence of this bacteria across all sites that were sampled in the communities.

**Conclusions/Significance:**

These data indicate the need for increased awareness of rodent-borne disease and the potential for environmental spread along the communities in areas undergoing significant land-use change.

## Introduction

Zoonotic pathogens account for more than 60% of the emerging infectious diseases that have arisen in the last 70 years [1]. Land-use changes, such as deforestation, agricultural expansion, and animal habitat disruption, are contributors to zoonotic transmission events [2], as these changes can lead to 1) perturbations in animal populations and the microbial communities that inhabit them and 2) increased animal-human interaction [2–4]. A prime example of a region that has undergone dramatic land disturbance is the southern Peruvian Amazon, where, in recent years, wide swaths of the landscape have quickly been converted for agricultural, logging, and mining purposes [5,6]. The completion of the Inter-Oceanic Highway that bisects this region of the Amazon has been a critical driver of these ecologic changes and has spurred a large influx of humans [6]. Thus, there is a need to survey the “pathogen landscape” in the new settlements along the highway to assess the communities’ risk for zoonotic infections [7].

In Peru, *Leptospira* and *Bartonella* are two prevalent yet understudied causes of bacterial zoonoses [8,9]. Pathogenic spirochetes of the genus *Leptospira* and at least 17 *Bartonella* species can cause fatal disease in humans in the absence of antimicrobial intervention [10,11]. Rapid detection of disease caused by these organisms is often impeded by the complexity of the laboratory diagnosis, especially in remote and resource-limited settings [10,11]. *Leptospira* and *Bartonella* are maintained primarily in small animal reservoirs, including domestic, peridomestic, and wild rodents [10,11]. *Leptospira* spirochetes are shed in the urine of animals, which may contaminate environmental water sources, including water used for drinking and sanitation, resulting in transmission to humans [11], whereas *Bartonella* infections in humans occur primarily through the bites of ectoparasitic arthropods or through scratches by infected animals [10,12].

Previous studies have shown that pathogenic *Leptospira* species are enzootic in the northern Peruvian Amazon [13,14], and hyperendemic levels of leptospirosis have been reported in the northern Andean and Amazonian regions of Peru [15–19], with diverse animal reservoirs and contaminated environmental sources having been identified [20–25]. Similarly, reports of bartonellosis in Peru date back to 1885, when Carrion’s disease (Oroya fever) was first described by Daniel Carrión [26], and endemic levels of *Bartonella* have been reported in the Andean and upper jungle regions of Peru [27]. A few studies have found dogs and ectoparasitic arthropods to be among the main carriers of *Bartonella* in these regions [28–31]. However, little is known about the prevalence of *Leptospira* or *Bartonella* reservoirs in the southern Peruvian Amazon. The results of 2 cross-sectional seroprevalence studies indicate that people living in the city of Puerto Maldonado and in Manu Province, both of which are in the southern Amazon state (departamento) of Madre de Dios, are highly exposed to *Leptospira* sp. (11% and 37% of individuals, respectively) [32,33]; however, no study has evaluated the rodent reservoir(s) or environmental sources that may pose a risk to the growing communities in these areas. A recent survey of inhabitants along the Inter-Oceanic Highway in Madre de Dios demonstrated that although it was widely recognized that rodents carry diseases, there was a lack of awareness among the inhabitants regarding their risk of acquiring those diseases [5]. Given the unknown zoonotic risk faced by the people in these communities and the challenges associated with public health awareness of zoonotic disease, we initiated studies to survey the prevalence of *Leptospira* and *Bartonella* species in rodents and sources of *Leptospira* in the environment around 4 communities along the Inter-Oceanic Highway in Madre de Dios.

## Methods

### Ethics statement

Approval for this study was obtained from the U.S. Naval Medical Research Unit No 6 (NAMRU-6) Institutional Animal Care and Use Committee. The experiments reported herein were conducted in compliance with the Animal Welfare Act and in accordance with principles set forth in the “Guide for the Care and Use of Laboratory Animals,” Institute of Laboratory Animal Resources, National Research Council, National Academy Press, 2011 and from the Peruvian Forestry and Wildlife Service (Servicio Nacional Forestal y de Fauna Silvestre [SERFOR]) (approval no. RD 0387-2012-AG-DGFFS/DGEFFS).

### Study sites and rodent sampling

Four communities along the Inter-Oceanic Highway in Madre de Dios were chosen for this study: Santa Rosa, Florida Baja, La Novia, and Alegria. The communities were selected based on the degree of man-made disturbance to the natural habitats; those chosen had both disturbed and non-disturbed areas. Six grids (area of 70 m^2^) with live-capture traps were placed in each community: 1 grid in disturbed areas (e.g. cattle grazing, pasture, cropland), 3 grids in edge areas (the borders between disturbed and relatively pristine forest), and 2 grids in non-disturbed areas. Each grid consisted of 7 lines with 7 trap stations each, giving a total of 61 traps, comprising 49 Sherman traps (H. B. Sherman Trap Company, Tallahassee, FL) and 12 Tomahawk traps (Tomahawk Live Trap Company, Tomahawk, WI). Trapped rodents were subjected to necropsy and tissues were collected, with cross-contamination between rodent dissections being minimized by using separate sterile sets of tweezers and scissors for each animal.

### Rodent species identification

Mammalogists from the Museo de Historia Natural, Universidad Nacional Mayor de San Marcos, Lima, identified rodent species in the field, taking morphologic measurements and photographs. All species determinations were subsequently confirmed by using taxonomic keys, measuring the skulls and jaws, and performing direct comparisons with the Collection of Mammals at the Museo de Historia Natural. If the morphologic identification of a species proved ambiguous, the mitochondrial gene encoding *cytochrome b* was amplified by PCR, using the primers MVZ 005 and MVZ 016 [34]. Amplicons were sequenced by Macrogen, Inc. (Seoul, Korea), and the sequence editing was performed using CodonCode Aligner v 6.0.2.

### Environmental sampling

We collected standing ground water during the dry season (September—October 2014), rainy season (January—February 2015), and mid season (April—May 2015). The average daily rainfall during the dry season is 40 mm, compared to at least 400 mm in the rainy season. When feasible, water samples were collected in 50-mL conical tubes from sites surrounding each grid and from areas frequented by persons living in each of the 4 study communities. During the dry and mid seasons or when standing water could not be readily found, soil samples were collected in 50-mL conical tubes at similar locations. Samples were stored at 4°C until processed 4 to 6 h later at the NAMRU-6 laboratory in Puerto Maldonado. Water samples were centrifuged at 8,000 × *g* for 40 min at 4°C, then the water supernatant was decanted and the remaining pellet stored at −80°C, along with the soil samples, until laboratory testing could be performed.

### Field processing of rodent urine and kidneys

Urine was aspirated from animal bladders during necropsy by using 1-mL syringes, and 1 or 2 drops were used to inoculate Ellinghausen-McCullough-Johnson-Harris (EMJH) medium containing 5-fluorouracil. The remainder (<100 μL) was placed in 1-mL cryovials for future laboratory testing. Animal kidneys were excised, and a quarter of each kidney was mashed up with sterile forceps then used to inoculate EMJH cultures. Each day’s EMJH cultures were transported from the field to the NAMRU-6 laboratory in Puerto Maldonado, where they were stored at ambient temperature (approximately 29–32°C) for 5 to 10 days before shipment to the main NAMRU-6 laboratory in Callao, outside Lima, where a 30°C bacteria incubator was available. Kidney and urine samples were stored at −80°C while awaiting shipment to Callao.

### *Leptospira* identification by culture-based and real-time PCR methods

EMJH cultures were examined weekly for leptospires by dark-field microscopy for up to 2 months after collection. DNA was extracted from soil and water sample pellets by using a MO BIO PowerSoil DNA Isolation Kit (QIAGEN). The remaining three-quarters of each animal kidney was homogenized with a mixer mill (Retsch), then DNA was extracted from the homogenate and from animal urine by using a MasterPure DNA Purification Kit (Epicentre). Real-time PCR (RT-PCR) with primers targeting the *lipL32* gene was used as an initial screen for pathogenic *Leptospira* species as previously described [35].

### Analysis of 16S metagenomic sequencing to identify *Leptospira* and *Bartonella* species

A series of 16S rRNA gene amplicon libraries were generated via PCR by using the NEXTflex 16S V1-V3 Amplicon-Seq Library Prep Kit (Bioo Scientific) in accordance with the manufacturer’s instructions. PCR products were cleaned with an AMPure XP PCR purification kit (Beckman Coulter) and then quantified by the Quant-iT PicoGreen assay (Illumina) and normalized by DNA concentration for sequencing. The samples were analyzed via paired-end sequencing with the Illumina MiSeq system. Total reads (read length: 2 × 300bp) were assessed by FastQC [36], with low-quality read bases (quality score <20) being trimmed by Trim Galore! (average read length: 228bp) [37]. Filtered reads were then subjected to taxonomic classification by the Kraken pipeline at the nucleotide level [38]. Our bacterial prevalence data were based on samples that yielded more than 500 classified reads, a cutoff that has been used with similar datasets [39]. Only samples with more than 5 reads that were classified as belonging to the *Leptospira* or *Bartonella* genera were considered positive [40]. As an additional means of reducing the reporting of false positives, *Leptospira-* and *Bartonella*-classified reads had to be present at 0.001% abundance for animals or 0.01% abundance for environmental samples, because the mean number of total reads for these different sample sets varied by a factor of 10 (36,742 vs. 258,708, respectively). All 16S metagenomic data have been deposited in the National Center for Biotechnology Information Short Read Archive under accession number SRP127615.

### Statistical analyses

Logistic regression analysis was used to assess differences in *Leptospira* and *Bartonella* positivity in rodent species and by season. Non-parametric proportion tests were used to assess differences in *Leptospira* positivity in environmental samples according to the level of habitat disruption. A *P*-value of <0.05 was considered statistically significant. For the correlation between bacteria and land disturbance analysis of the composition of microbiomes (ANCOM) [41] was employed, for each observed organism identified to the genus or species level, a log ratio of the species or genus count was calculated to determine which taxa are differentially abundant between samples from two different disturbance categories (e.g. disturbed, non-disturbed, edge, town) with p values undergoing the Bonferroni correction for multiple comparisons. Statistical analyses were performed with STATA statistical software, Version 13 (StataCorp) and R: A Language and Environment for Statistical Computing (R Foundation for Statistical Computing).

## Results

### Diverse mammalian species were sampled across varying levels of land perturbation

**Fig 1:**
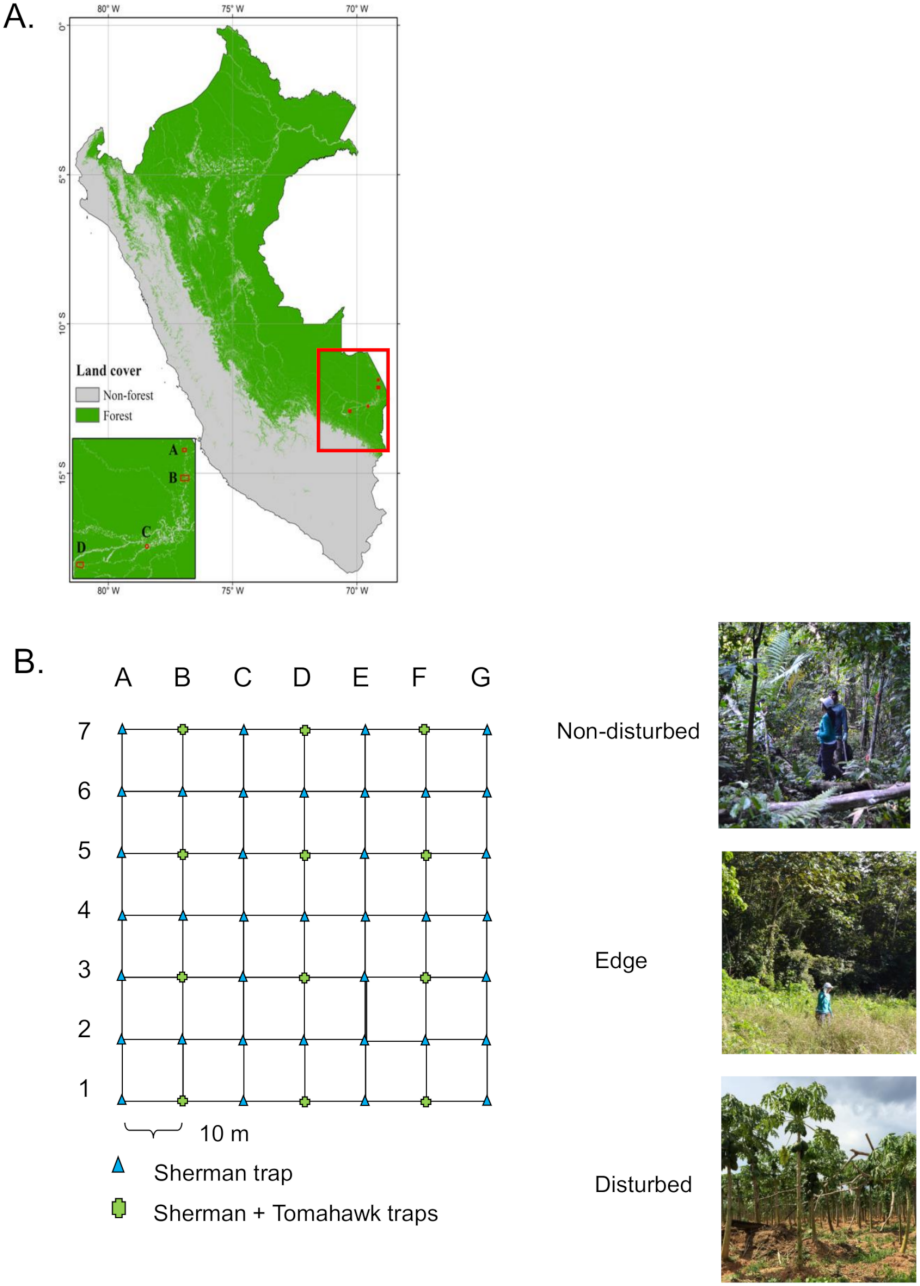
Rodent capture strategy in Madre de Dios, southern Peruvian Amazon. (A) Four communities along the Inter-Oceanic Highway were chosen to investigate the prevalence of rodent-borne disease: La Novia [A], Alegria [B], Florida Baja [C], and Santa Rosa [D]. (B) Sherman and Tomahawk traps were placed in 7×7 grids in areas with varying levels of habitat disruption, as shown in representative photos for non-disturbed, edge, and disturbed grids. **Map imagine made using data points collected by GPS devices and visualized in ArcGIS 10.0 (Esri)**.

We collected samples from 4 communities (Santa Rosa, Florida Baja, La Novia, and Alegria) along the Inter-Oceanic Highway (Fig 1A) during the dry season (September-October) and rainy season (January-February) in 2014–2015. By using a combination of Sherman and Tomahawk traps set across 7×7 grid formations placed at non-disturbed, edge, and disturbed sites (Fig 1B), we captured a total of 97 rodents. Comparable numbers of animals were captured during the rainy and dry seasons in each of the communities (Santa Rosa: 11 in the rainy season vs. 6 in the dry season; Florida Baja: 35 vs. 20; La Novia: 1 vs. 5; Alegria: 9 vs. 10). Most animals (n = 67, 69%) were trapped in “edge” grids that contained transitional areas at the borders between non-disturbed and disturbed lands (Table 1). Although fewer animals were caught in disturbed grids (n = 21) and non-disturbed grids (n = 9) (Table 1), we identified 10 different rodent species overall by morphologic examination and by sequencing their *cytochrome b* genes, and we observed greater species diversity in edge and non-disturbed areas than in disturbed areas (Table 1). *Oligoryzomys microtis* (the small-eared pygmy rice rat) was the most abundant species sampled (n = 69, representing 71% of the total animals). Approximately equal numbers of female and male rodents were sampled (representing 53% and 47%, respectively, of the total), and most (81%) of the animals caught were adults; only 8 juveniles and 11 subadults were caught (respectively representing 8% and 11% of the total).

**Table 1:** Species and distribution of animals caught in non-disturbed, edge, and disturbed grids.

		Grid Type			
		Non-disturbed	Edge	Disturbed	Total
Genus	Species				
*Euryoryzomys*	*nitidus*	**2**	**0**	**0**	**2**
*Holochilus*	*sciureus*	**0**	**1**	**0**	**1**
*Hylaeamys*	*perenensis*	**4**	**1**	**0**	**5**
*Neacomys*	*amoenus*	**0**	**1**	**0**	**1**
*Necromys*	*lenguarum*	**0**	**5**	**5**	**10**
*Oligoryzomys*	*microtis*	**1**	**54**	**14**	**69**
*Oxymycterus*	*inca*	**0**	**2**	**1**	**3**
*Proechimys*	*brevicauda*	**0**	**1**	**0**	**1**
*Proechimys*	*pattoni*	**1**	**0**	**0**	**1**
*Proechimys*	*simonsi*	**1**	**2**	**1**	**4**
	**Total n (%)**	**9 (9)**	**67 (69)**	**21 (22)**	

### *Bartonella* is more prevalent than *Leptospira* in rodents, and its prevalence does not vary across the rainy and dry seasons

**Fig 2:**
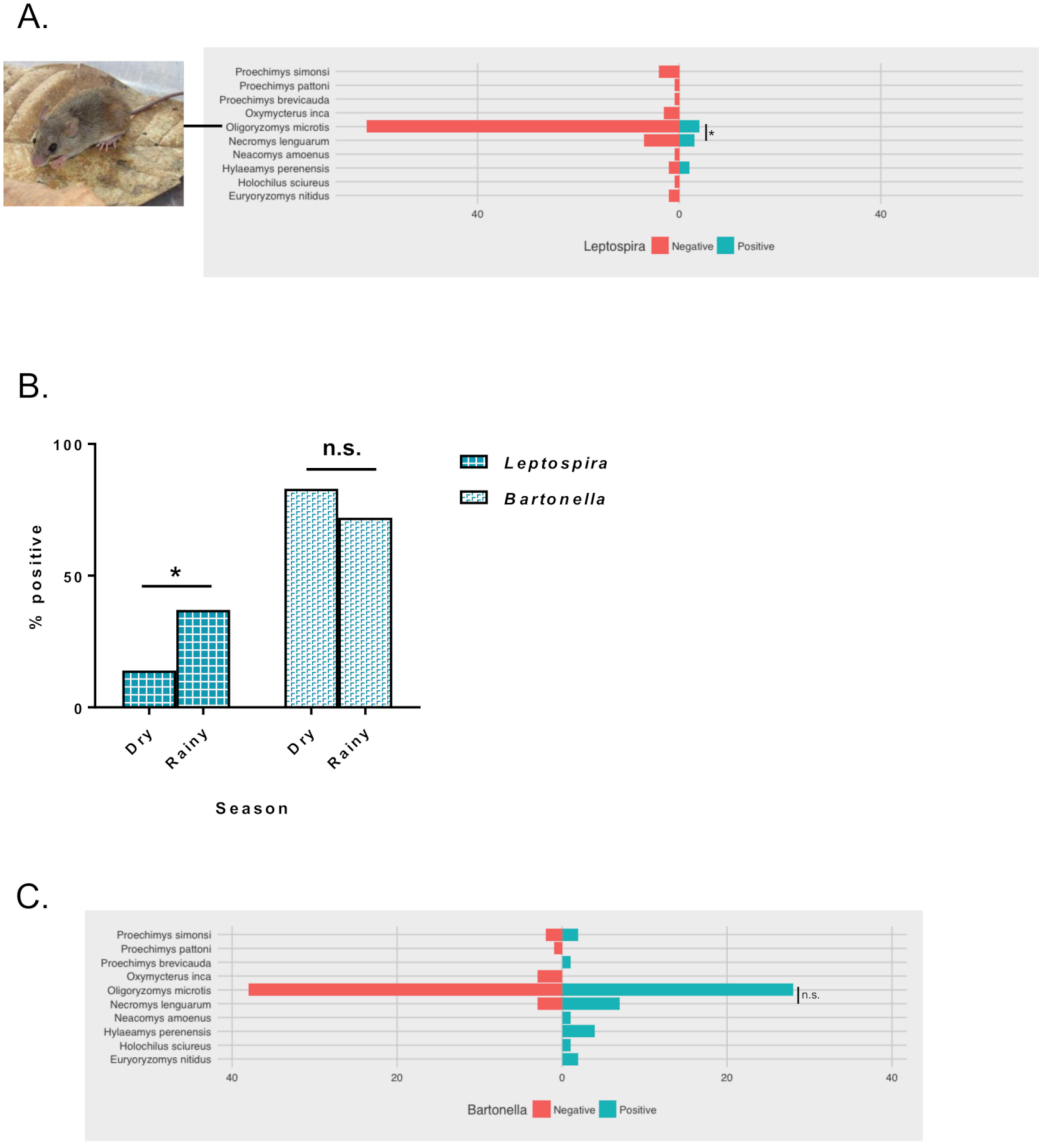
Molecular testing of rodent kidney DNA reveals varying patterns of *Leptospira* and *Bartonella* prevalence. The percent of rodents testing positive for *Leptospira* (A) and *Bartonella* (B) with statistical differences between the 2 most abundant species: *O*. *microtis* and *N*. *lenguarum* noted. The difference in prevalence across the rainy and dry seasons with statistical significance noted (C). Logistic regression used in both analyses. Significant, *; non-significant, n.s.

An initial screen for leptospires in EMJH cultures that were inoculated in the field with mashed kidney or aspirated urine yielded no growth after 2 months. We subsequently extracted DNA from all 97 kidney samples and 38 urine samples, then we used 2 molecular methods to test for the presence of *Leptospira*. First, by using primers targeting the pathogenic *Leptospira* gene *lipL32*, we detected *Leptospira* in 6 animals (6%), including 2 for which a urine sample also tested positive. Next, to ensure that we were not missing any *Leptospira*-positive animals and to identify other bacterial species that might be present, we performed 16S metagenomic sequencing. Samples for which a sufficient amount of DNA remained (n = 93) yielded between 23 and 308,943 reads per sample. Operational taxonomic unit (OTU) picking with Kraken [38] indicated that 5% to 100% of reads could be classified for each sample. From among those samples that yielded more than 500 classified reads (n = 72), we identified an additional 17 animals that tested positive for *Leptospira*, bringing the total to 23 out of 97 animals (24%). As regards the 2 species for which more than 10 individual animals were tested, i.e., *Necromys lenguarum* (the Paraguayan bolo mouse) and *O*. *microtis*, *N*. *lenguarum* mice were more likely to test positive for *Leptospira* than were *O*. *microtis* rats (OR = 5.27, 95% CI: 1.30–21.32, *P* = 0.02) (Fig 2A). Overall, animals were more likely to test positive for *Leptospira* in the rainy season (n = 15/41; 37%) than in the dry season (n = 8/56; 14%) (OR = 3.46, 95% CI: 1.30–9.24, *P* = 0.01) (Fig 2B). Although *Leptospira* infections were detected in similar proportions of female and male animals (25% vs. 22%), there appeared to be more cases in adults (n = 20/78; 26%) than in subadults (n = 2/11; 18%) or juveniles (n = 1/8; 13%).

The OTU results from the 16S metagenomic analysis also identified a large proportion of animals that were positive for *Bartonella* species (n = 56/72; 78%). Compared to the seasonal trend found with *Leptospira*-positive animals, a slightly lower proportion of *Bartonella*-positive animals were detected during the rainy seasons (n = 26/36; 72%) than during the dry seasons (n = 30/36; 83%), but this difference was not statistically significant (OR = 1.92, 95% CI: 0.61–6.01, *P* = 0.26) (Fig 2B). Among the most abundant species tested, *N*. *lenguarum* mice were more likely to test positive for *Bartonella* than were *O*. *microtis* rats; however, this was not statistically significant (OR = 3.24, 95% CI: 0.37–28.22, *P* = 0.29) (Fig 2C). There was no difference in the *Bartonella* prevalence in female and male animals (77% and 79%, respectively), and the proportions of *Bartonella*-positive individuals were also similar for adults (n = 47/57; 82%) and subadults (n = 6/8; 75%), whereas a smaller proportion of juveniles tested positive (n = 3/7; 43%).

Co-infection with both *Leptospira* and *Bartonella* was detected in 19 animals; only 4 animals were positive for *Leptospira* alone.

### 16S metagenomic sequencing highlight indicator bacterial species across habitat disruption

**Fig 3:**
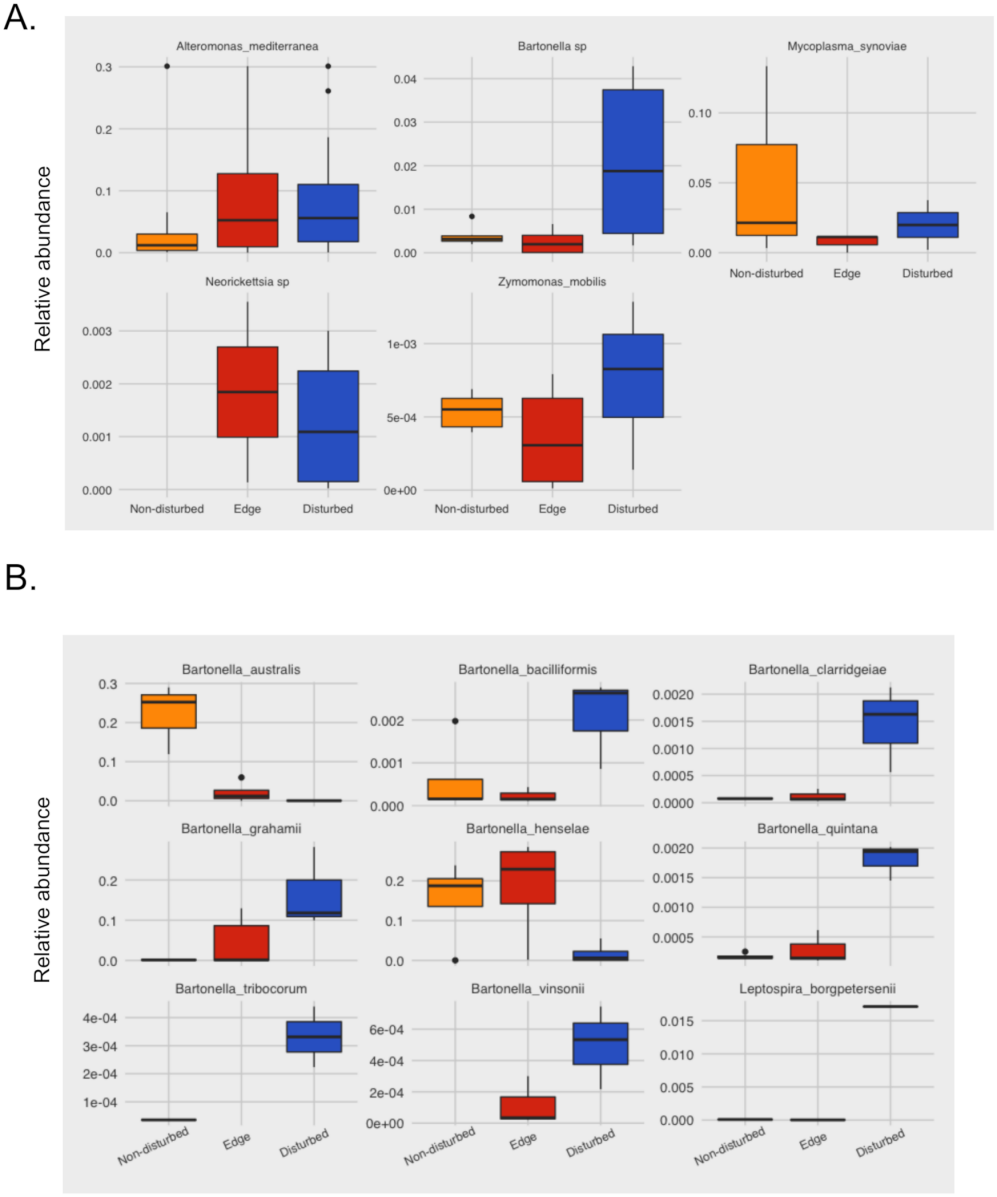
Analysis of microbial composition in rodent samples relative to land disturbance. (A) Abundance of 5 bacteria that correlate with grid type. (B) OTU classified at the species level and relative abundance of *Bartonella* and *Leptospira* sp. distributed across land disturbance.

Given that there was an uneven distribution of animals captured across grids and in some cases inadequate sample sizes for comparison, we next took a reverse approach to determine whether there were bacterial signatures that could indicate whether the level of land disturbance is amenable to rodents that are colonized by zoonotic bacteria. Using a method that was generated for the analysis of microbiomes (ANCOM), we identified 5 bacteria (*Alternomonas mediterranea*, *Zymomonas mobilis*, *Mycoplasma synoviae*, *Bartonella* sp. and *Neorickettsia* sp.) whose relative abundance was significantly correlated to the level of land disruption (Fig 3A). From this group of 5 bacteria, both *Bartonella* sp. and *Neorickettsia sp*. have high zoonotic potential and are important to human health. While *Bartonella* abundance was associated with increasing levels of land disturbance, whereas *Neorickettsia* trended in the opposite direction, with higher abundance seen in edge areas compared to disturbed areas.

A closer examination of OTU and relative abundance for *Bartonella* across grid type revealed that multiple species were classified, including *B*. *australis*, *B*. *bacilliformis*, *B*. *clarridgeiae*, *B*. *grahamii*, *B*. *henselae*, *B*. *quintana*, *B*. *tribocorum* and *B*. *vinsonii* (Fig 3B). While the trends across the grids differed according to species, in a few cases with higher abundance in non-disturbed areas compared to disturbed, only the OTU classified to the *Bartonella* genera were significantly associated with the level of land disturbance. Although OTUs for *Leptospira* were not identified as signatures of land disturbance in our analysis, we noted *L*. *borgpetersenii* (the sole *Leptospira* sp. classified to species level) in disturbed areas (Fig 3B).

### A high prevalence of *Leptospira* was found in the environment across all sites and seasons

**Fig 4:**
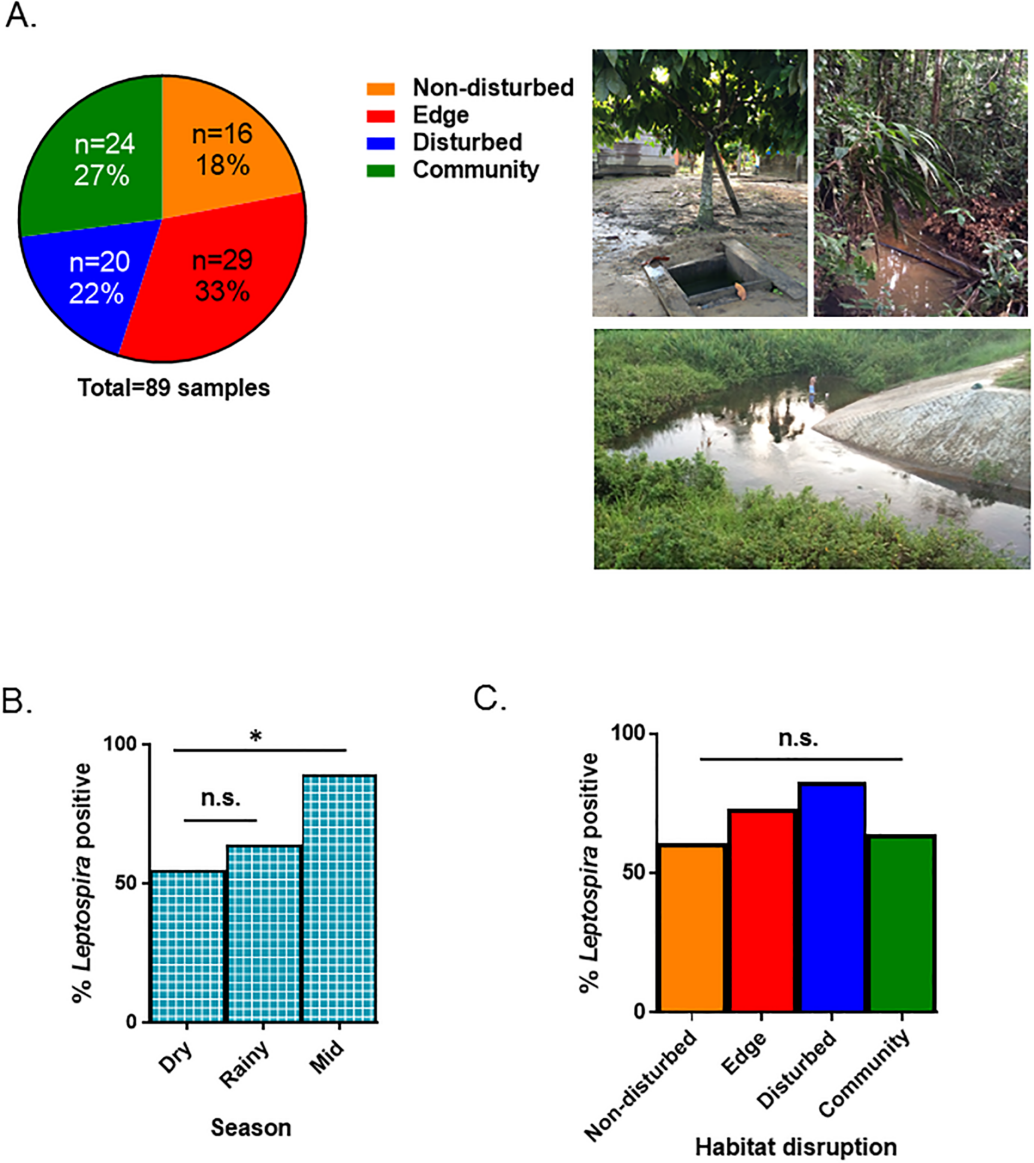
Summary of environmental samples collected and *Leptospira* positivity identified by molecular testing. (A) The overall distribution of samples by location and representative photos that show the varying environments that were sampled. *Leptospira* positivity according to season (B) and level of habitat disruption (C), with statistical significance noted. Logistic regression and non-parametric proportions tested were used, respectively. Significant, *; non-significant, n.s.

A total of 89 environmental samples were collected across 3 seasons: 21 and 38 samples, respectively, were obtained during the same dry and rainy seasons in which the rodent trappings were carried out, and an additional 30 samples were collected during the mid season (April—May 2015). Combining the samples from all 3 collecting trips, 16 samples in total were collected from disturbed areas, 29 from edge areas, 20 from non-disturbed areas, and 24 at locations in or around the communities. Samples of tap water at the mobile laboratory sites and at the main laboratory in Puerto Maldonado served as negative controls (Fig 4A). In areas or seasons in which water was unavailable for sampling, a soil sample was collected in places where standing water had existed previously or at the entrance to the grid. Overall, 64 (72%) of the 89 samples were from water sources and the remaining 25 samples (28%) were collected from soil; however, only water samples were screened for bacterial growth in EMJH cultures.

After approximately 1 month in culture, only a single water sample, collected from the hostel (hospedaje) where the field team stayed in the community of Santa Rosa, yielded detectable leptospires that were visible by dark-field microscopy. However, given that both pathogenic and nonpathogenic species of *Leptospira* can survive in the environment [21], we employed an RT-PCR assay with primers targeting *lipL32* to determine whether this isolate was a pathogenic strain. The results of the RT-PCR assay indicated that the leptospires detected were nonpathogenic and, thus, the sample was categorized as negative. We subsequently extracted DNA from all 89 environmental samples, 9 of which tested positive (10%) by *lipL32* RT-PCR. We used 16S metagenomic sequencing to screen all these samples and obtained DNA libraries suitable for sequencing from 63 (71%) of them. For each sample, between 7,422 and 622,049 reads were generated, of which between 98% and 99% were classified by Kraken [38]. In this group of samples, an additional 35 tested positive for *Leptospira*, bringing the total number of positives to 44 out of the 89 total samples (49%). Of these 44 positive samples, approximately equal numbers were derived from soil (n = 21; 48%) and from water (n = 23; 52%). A slightly smaller proportion of samples collected during the dry season tested positive (n = 6/11; 55%) when compared with samples collected during the rainy season (n = 21/33; 64%; p = 0.60) or mid season (n = 17/19; 89%; p = 0.04) (Fig 4B). *Leptospira* was detected in a relatively high proportion of samples from every level of habitat perturbation and from areas around the towns (in 61%–83% of samples), and these differences were not statistically significant (Fig 4C). None of the water samples (n = 3) collected from running taps in the community or at the laboratory in Puerto Maldonado tested positive for *Leptospira*. As we had species level classification of *L*. *interrogans*, *L*. *borgpetersenii*, and *L biflexa*, we did a subset analysis of prevalence removing nonpathogenic *L*. *biflexa*. One caveat is that some samples (n = 3) contained bacteria that classified solely as *Leptospira* sp., which may represent a pathogenic or nonpathogenic strain that could bias this analysis.

## Discussion

Punctuated by the completion of the Inter-Oceanic Highway in the state of Madre de Dios, the southern Peruvian Amazon has undergone significant land-use change that has resulted in a large increase in the number of human settlements in the region. This study represents an initial effort by our group toward assessing the risk of bacterial zoonotic disease in these communities. We focused on the rodent-borne reservoir of *Leptospira* and *Bartonella*, as well as on potential environmental sources of human exposure to *Leptospira* in the 4 selected communities along the Inter-Oceanic Highway.

Overall, we identified 11 species of rodents and found a high prevalence of *Bartonella* (78%) and a moderate prevalence of *Leptospira* (24%), identified primarily by 16S metagenomic sequencing. Although we attempted other methods of *Leptospira* detection, including culture and pathogen-specific PCR directly from samples, these approaches yielded only limited results because of challenges encountered in the field and laboratory. Our use of 16S metagenomic sequencing was inspired by 2 studies that used that technique to screen for rodent-borne bacterial zoonoses. The first of these studies, conducted in Senegal, found a low (20%) prevalence of *Bartonella* in a survey of commensal rodents that included species from 3 genera (*Rattus*, *Mastomys*, and *Mus*) [39], whereas the second study, conducted in France, found a high (89%) prevalence of *Bartonella* in voles but a much lower (2%) prevalence of *Leptospira* [40]. The differences in prevalence could be explained by differences in the animal populations investigated, as well as in the geographic locations, habitats surveyed, and seasonal timing of collections. A study conducted in the northern Peruvian Amazon near Iquitos, Peru, in the Loreto department, where the habitat and species richness are more comparable to those of Madre de Dios in the southern region of Peru, found a 20% prevalence of *Leptospira* in wild rodents [25], which was comparable to the 24% prevalence detected in our study. Similarly, another study reported a 21% prevalence in wild rodents captured in south central Chile [42]. Given that *Leptospira* is endemic to both the northern Peruvian Amazon and south central Chile, the results of our study suggest that this organism is also endemic to the southern Peruvian Amazon. Animal reservoirs for *Bartonella* in Peru have remained elusive, although a single study in La Convención Province, which is adjacent to Madre de Dios, implicated small rodents as a potential sources of *Bartonella* in the region [43]. However, that study of 28 animals found a prevalence of only 17.9%, which is much lower than that found in our study and might be explained by the species encountered in the earlier study, namely *Rattus rattus* (the black rat) and a few examples of *H*. *perenensis* (the western Amazonian oryzomys) and *Oecomys* sp. Still, these data lend evidence to our study that implicates wild rodent species as a potential reservoir of *Bartonella* sp.

A high prevalence of *Bartonella* was identified in animals captured across both trapping seasons, whereas *Leptospira* positivity was higher during the rainy season than during the dry season. We also observed a seasonal fluctuation in the presence of *Leptospira* in the environments of these communities, as has been previously reported [44], but the differences we observed were only significant between the dry and mid season. However, it is notable that we found such a high prevalence across all the sites that we sampled, including within the communities, because this prevalence was on a par with that in an urban slum (47%) and was higher than that in a similarly rural area in the northern Peruvian Amazon (25%) [20]. These differences could be explained by several factors, including the method of detection, the season during which the samples were collected, and the type of samples. In our study, we included soil samples in lieu of water if the latter was not available. As *Leptospira* can remain in soil for up to 5 months, depending on the environmental conditions [45], this approach may have increased our ability to detect the pathogen in the environment. Finally, although we did not have the species resolution to determine the relationship between *Leptospira* in wild rodents and that in the environment, previous studies have shown that these factors may not correlate [46], given that domestic animals can also serve as a bridge between rodent and environmental reservoirs.

The results of our study should be interpreted in the context of its strengths and weaknesses. First, our trapping strategy during the rainy and dry seasons, as well as across different levels of habitat disruption provided us with a diverse set of samples to analyze. While we leveraged the animal trappings from 6 grids in 4 communities with sequential sampling, these efforts only yielded a total of 97 animals. Accordingly, we made the best use of the data by reporting observed trends and performing statistical analyses only when the sample sizes were adequate. We can still only speculate as to which species of rodents are especially amenable to colonization by bacterial pathogens and their likelihood to be transmitted given their proximity to humans. Additional studies of zoonotic pathogens in rodent species across varying habitats are ongoing by our group and will include a multiyear dataset with a larger sample size, thus relieving any limitations on sample size. Still, our dataset yielded 16S sequencing that enabled us to identify 5 indicator species, including *Bartonella* and *Neorickettsia*, that correlated with land disturbance and therefore may be related to the rodent species that are inhabited by these microbes. Second, as discussed by Galan et al. [39], a major limitation of metagenomic sequencing is the inability to determine whether an OTU is derived from a pathogenic or nonpathogenic bacterial species. To partially address this issue, our 16S dataset analyzed the V1–V3 gene region and, therefore, could in theory be used to classify reads at the species level [47]. We took note of any sequences that mapped to the sole nonpathogenic *Leptospira* strain that was present in our environmental samples, *L*. *biflexa*, and excluded these reads when calculating our prevalence data. However, for the majority of samples the 16S resolution did not reach the species level, so we cannot be certain that each sample contained pathogenic bacteria and this prevented us from compiling a complete list of *Leptospira* and *Bartonella* species that circulate among these communities in Madre de Dios. Our attempts to overcome this limitation by obtaining live cultures was also met with challenges, including suboptimal field conditions and limited resources, which prevented us from verifying and identifying the specific species present in our samples. Nevertheless, our study was strengthened by using a *lipL32* real-time PCR to identify pathogenic strains in our samples as well as the sensitive, high-throughput metagenomic sequencing approach that yielded results faster than pathogen-specific PCR methods and allowed us to look at a greater breadth of bacterial species that may or may not be capable of isolation in culture (an analysis of which will generally underestimate the true prevalence). The application of this methodology to future surveillance studies for *Leptospira* and *Bartonella* may improve the detection of these often-elusive pathogens, thereby enabling a more accurate assessment of their prevalence across regions. Further, ANCOM results for the bacteria-rich water and soil samples showed that a subset of bacterial species (unrelated to human health) correlate with land disturbance, and thus could be used to study the ecological impact of deforestation and encroachment of human settlements that are rapidly transforming the microbial landscape in biodiverse settings (S1 Fig). However, the feasibility of such an approach would depend on the laboratory capacity and computing power to analyze the sequence data. While validation with pathogen-specific RT-PCR may still be necessary for delineating pathogenic from non-pathogenic species, advances in sequencing technologies and the generation of longer reads may eventually allow for greater species resolution in the near future.

In summary, this is the first study to use 16S metagenomic sequencing to determine the prevalence of *Leptospira* and *Bartonella* species and to investigate what could be a growing risk of transmission in a region of the world undergoing rapid land-use change. Given the potential for spillover from these sources into the human populations in these areas, our data highlight the importance of studying the impact of habitat perturbation on animal and human health, which will require additional surveillance and risk assessments to inform public health prevention measures. Critically, these data demonstrate the need for increased awareness of rodent-borne disease, including both animal and environmental sources, among the communities of people living in the area, as well as among health care providers. We are presently working to extend these initial findings to additional zoonotic pathogens in order to make recommendations for comprehensive public health strategies that may be applied in the southern Peruvian Amazon and in other areas of high habitat disturbance.

## Supporting information

S1 Fig Analysis of microbial composition in samples from the environment relative to land disturbance. Abundance of bacteria that correlate with land disturbance in the (A) soil and (B) water.
(TIF)
