# Supplementary figures and images for: Identification of Leptospira and Bartonella among rodents collected across a habitat disturbance gradient along the Inter-Oceanic Highway in the southern Amazon Basin of Peru
Source: PLoS One. 2018 Oct 9;13(10):e0205068. doi: 10.1371/journal.pone.0205068 (PMC6177132; doi:10.1371/journal.pone.0205068)

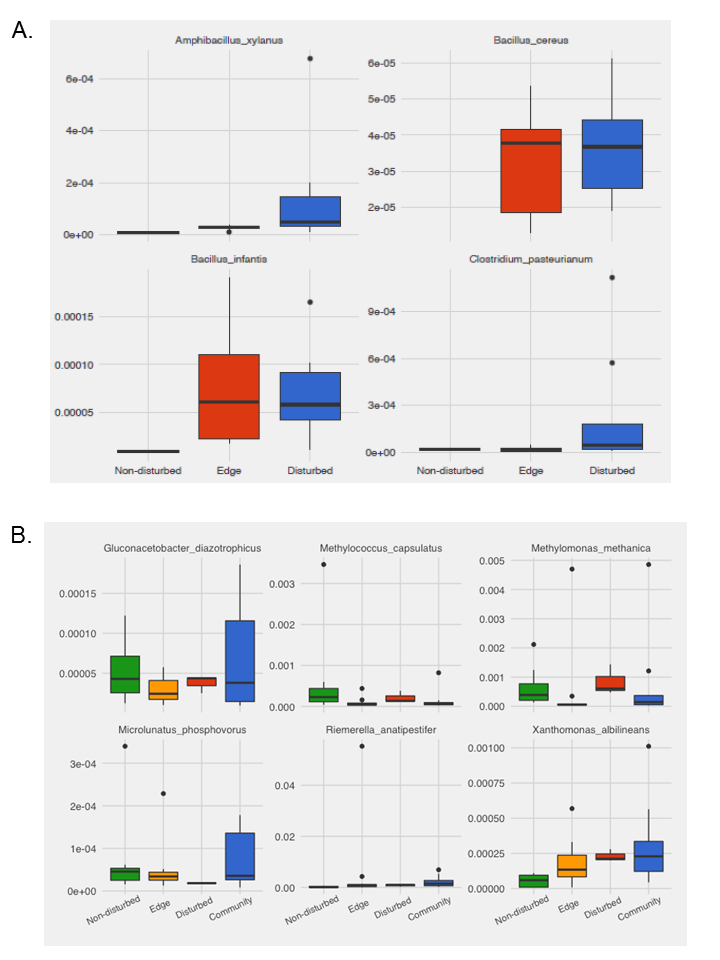

Supplement: S1 Fig — Abundance of bacteria that correlate with land disturbance in the (A) soil and (B) water. (TIF) [file pone.0205068.s001.TIF]
